# Adiposity and Cumulative Cardiometabolic Risk Factors as Determinants of Hepatic Steatosis Severity in Postmenopausal Women

**DOI:** 10.3390/jcm15156098

**Published:** 2026-08-05

**Authors:** Ifigeneia Avgousti, Areti Augoulea, Eleni Armeni, Dimitrios S. Karagiannakis, Sofia Paraskevopoulou, Konstantinos Panoulis, Sofia Kalantaridou, Nektaria Papanikola, Anastasia Angelopoulou, George Papatheodoridis, Makarios Eleftheriadis, Irene Lambrinoudaki

**Affiliations:** 1 2nd Department of Obstetrics and Gynecology, National and Kapodistrian University of Athens, Aretaieio Hospital, 11528 Athens, Greece; ifigeneia.avg@gmail.com (I.A.); aretiaugoulea@yahoo.gr (A.A.); elenaarmeni@hotmail.com (E.A.); panouliskonstantinos@gmail.com (K.P.); npapanikola@gmail.com (N.P.); a.angelopoulou10@gmail.com (A.A.); makarios@hotmail.co.uk (M.E.); 2 4th Department of Internal Medicine, National and Kapodistrian University of Athens, Attikon University Hospital, 12462 Athens, Greece; dkarag@med.uoa.gr (D.S.K.); sofiprsk@gmail.com (S.P.); 3 3rd Department of Obstetrics and Gynecology, National and Kapodistrian University of Athens, 12462 Athens, Greece; sophiakalantaridou@gmail.com; 4 1st Department of Gastroenterology, Medical School of National and Kapodistrian University of Athens, General Hospital of Athens “Laiko”, 11527 Athens, Greece; gepapath@med.uoa.gr

**Keywords:** postmenopausal women, steatosis, MASLD, cardiometabolic risk factors, body mass index, waist circumference, mid-upper arm circumference, sex hormone binding globulin

## Abstract

**Background/Objectives:** Postmenopausal women are at increased risk of metabolic dysfunction-associated steatotic liver disease (MASLD). This study evaluated the associations of anthropometric and hormonal parameters and cardiometabolic risk-factor accumulation with steatosis and MASLD. **Methods:** In this cross-sectional study, 87 postmenopausal women at a university menopause clinic underwent anthropometric, biochemical, and liver imaging assessment. Hepatic steatosis was defined as CAP ≥ 248 dB/m on transient elastography, and MASLD as steatosis with ≥1 cardiometabolic risk factor (2023 consensus criteria). Anthropometry included body mass index (BMI), waist circumference, mid-upper arm circumference, and triceps skinfold thickness. **Results:** CAP-defined steatosis (46.0%) closely mirrored MASLD (44.8%; all but one steatotic woman met ≥ 1 criterion). Waist circumference independently correlated with steatosis (OR = 2.77, 95% CI 1.08–7.13, *p* = 0.035), adding value beyond BMI (Nagelkerke R^2^ 0.202 vs. 0.140). Steatosis burden rose progressively with risk-factor number: each additional factor conferred a 30.59 dB/m CAP increase (95% CI 17.33–43.85, *p* < 0.001) and 3.38-fold higher odds of steatosis (95% CI 1.59–7.20, *p* = 0.002; ≥3 vs. 2 factors, Welch’s *p* = 0.004). **Conclusions:** MASLD and CAP-defined steatosis were largely coincident. Adiposity correlated with steatosis, but severity was determined chiefly by the accumulation of cardiometabolic risk factors, which is a key determinant of steatotic liver disease severity after menopause.

## 1. Introduction

The postmenopausal transition is characterised by profound hormonal changes that contribute to adverse cardiometabolic remodelling, including increased visceral adiposity, insulin resistance, and the heightened risk of metabolic dysfunction-associated steatotic liver disease (MASLD) [1,2,3]. These changes place postmenopausal women at a disproportionately increased risk of hepatic steatosis and its metabolic sequelae [2,3].

Adiposity is a central driver of MASLD; however, traditional measures such as body mass index (BMI) may inadequately capture the complex redistribution of fat that occurs after menopause [4,5]. In particular, central adiposity and ectopic fat accumulation are more strongly linked to metabolic dysfunction than overall body mass [6]. Alternative anthropometric measures, including waist circumference, mid-upper arm circumference (MUAC), and triceps skinfold thickness (TSF), may better reflect regional fat distribution, yet their relative contribution to MASLD risk in postmenopausal women remains poorly defined [7,8].

In parallel, sex hormone alterations, particularly reduced oestrogen and changes in sex hormone-binding globulin (SHBG), have been implicated in the pathophysiology of hepatic steatosis [9]. However, it remains unclear whether these hormonal factors exert independent effects or primarily reflect underlying adiposity-driven metabolic dysfunction in this population [10,11].

Despite the growing recognition of MASLD, evidence specifically focusing on postmenopausal women is limited, and the relative importance of central versus peripheral adiposity and hormonal factors in this group has not been fully elucidated [11,12,13].

Beyond individual adiposity or hormonal markers, MASLD is by definition a disease of coexisting cardiometabolic dysfunction, requiring hepatic steatosis together with at least one cardiometabolic risk factor [14]. After menopause, obesity, dyslipidaemia, hypertension and disordered glucose metabolism frequently cluster, so that affected women commonly carry several risk factors simultaneously [4]. Whether the burden of steatosis scales with the sheer number of accumulated cardiometabolic risk factors, rather than with any single adiposity or hormonal measure in isolation, has received little attention in postmenopausal cohorts; yet, it could have direct implications for how risk is stratified in this group [15].

Therefore, this study aimed to evaluate the associations of anthropometric measures and sex hormones with hepatic steatosis and MASLD in postmenopausal women, and to determine whether the accumulation of cardiometabolic risk factors is associated with the severity of hepatic steatosis in this population.

## 2. Materials and Methods

### 2.1. Study Population

This cross-sectional exploratory study was nested within a larger cohort of postmenopausal women attending the Menopause Clinic of the Second Department of Obstetrics and Gynaecology, National and Kapodistrian University of Athens, Greece, between October 2023 and February 2025, for first clinical assessment [16]. The clinic is a tertiary referral centre providing the management of climacteric symptoms and preventive counselling for long-term cardiometabolic and bone health.

Among the 490 women who provided written informed consent to participate in the cardiometabolic assessment programme, only those who additionally consented to undergo liver imaging for the evaluation of possible hepatic steatosis were included in the present analysis. A total of 114 women underwent liver imaging assessment, of whom 27 were excluded because of incomplete biochemical data required for the predefined analyses, resulting in a final analytical sample of 87 women. No a priori sample size calculation was performed as this is an exploratory, hypothesis-generating analysis of all cases available.

Menopause was defined as ≥12 consecutive months of amenorrhoea. Eligible participants were postmenopausal women aged ≥ 40 years. Exclusion criteria included history of malignancy, autoimmune hepatitis, recent antiviral therapy (≤6 months), untreated thyroid disease, acute or chronic inflammatory disorders, and excessive alcohol consumption (>20 g/day or >140 g/week), according to WHO criteria [17]. The study was approved by the local Institutional Review Board (approval code: 518/30-05-2023).

### 2.2. Clinical and Laboratory Evaluation

Medical history and lifestyle data were collected for all participants. Anthropometric measurements included weight, height, BMI, waist circumference, MUAC, and TSF, measured on the non-dominant arm using standardised techniques [18].

Fasting blood samples (≥8 h) were obtained to measure glucose, lipid profile, liver enzymes—namely aspartate aminotransferase (AST), alanine aminotransferase (ALT), and γ-glutamyl transferase (GGT)—as well as albumin, bilirubin, insulin, total testosterone, and sex hormone-binding globulin (SHBG). Biochemical assays were performed using the ARCHITECT c8000 system (Abbott Diagnostics, Abbott Park, IL, USA). Serum glucose was measured using the hexokinase/glucose-6-phosphate dehydrogenase method (coefficient of variation [CV] ≤ 5%). Insulin was measured using the ARCHITECT i1000 analyser (Abbott Diagnostics, Abbott Park, IL, USA); the assay had a CV ranging from 1.9% to 5.2% and an analytical sensitivity of 1 μIU/mL. Total testosterone and SHBG were measured using chemiluminescent microparticle immunoassays on the ARCHITECT i1000SR analyser (Abbott Diagnostics, Abbott Park, IL, USA). For the ARCHITECT 2nd Generation Testosterone assay, the within-laboratory CV ranged from 2.6% to 5.2%, with a limit of detection of 0.10 nmol/L and a limit of quantification of 0.15 nmol/L. For the ARCHITECT SHBG assay, total CVs were ≤14.6%, and the analytical sensitivity was 0.02 nmol/L. The free androgen index was calculated as [total testosterone (nmol/L)/SHBG (nmol/L)] × 100. Insulin resistance was estimated using the homeostatic model assessment of insulin resistance (HOMA-IR), calculated as [fasting insulin (μIU/mL) × fasting glucose (mg/dL)]/405.

### 2.3. Physical Activity Assessment

Physical activity was assessed using the International Physical Activity Questionnaire (IPAQ) [19], validated in the Greek population [20]. Weekly metabolic equivalent (MET) minutes were calculated. Participants were categorised as having high, moderate, or low physical activity according to established IPAQ scoring protocols; those not meeting moderate or high criteria were classified as sedentary.

### 2.4. Spectrum of Liver Disease Assessment

Hepatic steatosis was assessed using transient elastography (FibroScan^®^ 502 Touch, Echosens, Paris, France) via the controlled attenuation parameter (CAP). Measurements were performed by trained operators blinded to clinical data. A minimum of 10 valid measurements with an interquartile range (IQR)/median ≤ 30% was required, and mean values were used for analysis.

MASLD was defined according to the 2023 international consensus as the presence of hepatic steatosis together with at least one cardiometabolic risk factor. In this study, steatosis was defined as CAP ≥ 248 dB/m. CAP ≥ 248 dB/m was used because it is the value documented as equivalent to at least mild (≥S1) hepatic steatosis across a number of validation studies, including the individual patient-data meta-analysis of CAP measurements by Karlas et al. and subsequent non-invasive MASLD assessments [21,22]; although alternative cut-offs such as 236 dB/m have been proposed, 248 dB/m was adopted for consistency with these validation studies.

### 2.5. Statistical Analysis

Data were analysed using SPSS version 29.0. Continuous variables are presented as mean ± standard deviation and categorical variables as frequencies (%). The normality of the distribution was assessed using the Shapiro–Wilk test and also inspected visually. Not normally distributed values were log-transformed where possible to improve the characteristics of the distribution. Between-group comparisons were performed using independent-sample t-tests for continuous variables when normally distributed—Mann–Whitney U if the variables were not normally distributed and χ^2^ or Fisher’s exact tests for categorical variables.

In participants with liver imaging, Pearson correlation analyses assessed associations between clinical, anthropometric, and hormonal variables with hepatic steatosis (CAP).

Multivariable linear regression models were used to identify independent predictors of liver fat, with variables selected based on biological plausibility. Model fit was assessed using adjusted R^2^, and results are reported as unstandardised coefficients (B) with *p*-values. Additional models including BMI were constructed to explore attenuation of regional adiposity effects.

Multivariable regression analyses examined factors associated with MASLD and CAP-defined steatosis. Parsimonious models containing a limited number of pre-specified covariates were constructed because of the small number of outcome events and to reduce the risk of overfitting. To minimise multicollinearity, strongly correlated adiposity measures were not entered into the same model; mid-upper arm circumference and triceps skinfold thickness were therefore modelled separately alongside waist circumference and SHBG. Insulin and HOMA-IR, although the strongest univariable correlates of CAP, were not entered into the multivariable models because insulin resistance lies on the causal pathway between adiposity and hepatic steatosis, so their inclusion would represent over-adjustment; waist-to-hip ratio was not entered because it showed no univariable association with CAP. Tolerance and variance inflation factors were within acceptable limits for all reported models. Results are presented as odds ratios (OR) with 95% confidence intervals. Statistical significance was defined as *p* < 0.05.

## 3. Results

### 3.1. Clinical and Anthropometric Characteristics According to CAP-Defined Steatosis

A total of 87 postmenopausal women were included in the analysis (Table 1a). Women with CAP-defined hepatic steatosis (CAP ≥ 248 dB/m) did not differ in age or years since menopause compared to those without steatosis. However, they exhibited significantly higher adiposity, including body mass index (27.3 ± 4.7 vs. 24.5 ± 3.1 kg/m^2^, *p* = 0.003) and waist circumference (92.4 ± 12.5 vs. 84.3 ± 9.9 cm, *p* = 0.001).

No significant differences were observed in systolic or diastolic blood pressure. Triceps skinfold thickness and mid-upper arm circumference were higher in the steatosis group, with borderline statistical significance (*p* = 0.06 and *p* = 0.05, respectively). The prevalence of current smoking did not differ between groups (*p* = 0.116).

### 3.2. Hormonal Parameters According to MASLD Status

MASLD and CAP-defined steatosis identified almost the same women: 39 of the 40 women with steatosis met at least one cardiometabolic criterion, so the two groupings differed by a single participant. Comparisons stratified by either definition were therefore materially identical, and hormonal parameters are presented by MASLD status. As shown in Table 1b, women with MASLD had significantly lower SHBG concentrations compared to non-MASLD individuals (62.2 ± 39.4 vs. 83.5 ± 27.6 nmol/L, *p* = 0.009). Total testosterone, compared on log-transformed values, did not differ between groups (*p* = 0.850).

### 3.3. Correlates of Liver Fat (CAP)

Correlation analyses (Table 2) demonstrated that CAP was positively associated with body mass index (r = 0.508, *p* < 0.001), waist circumference (r = 0.433, *p* < 0.001), mid-upper arm circumference (r = 0.350, *p* = 0.002), and triceps skinfold thickness (r = 0.351, *p* = 0.002). CAP also correlated with the metabolic variable glucose (r = 0.317, *p* = 0.003), insulin (r = 0.609, *p* < 0.001), and HOMA-IR (r = 0.636, *p* < 0.001), which were the strongest univariable correlates of all the variables examined.

An inverse correlation was observed between CAP and SHBG (r = −0.254, *p* = 0.030). No significant associations were identified with age, years since menopause, blood pressure, or waist-to-hip ratio. Waist-to-hip ratio was not included in multivariable modelling because no significant association with CAP was observed in univariable analyses. Although insulin and HOMA-IR showed the strongest univariable associations with CAP, they were not entered into the multivariable models: insulin resistance lies on the causal pathway between adiposity and hepatic steatosis, so its inclusion would represent over-adjustment, and the limited number of outcome events precluded expanding the models with additional metabolic covariates without overfitting.

### 3.4. Multivariable Linear Regression Analysis

In multivariable linear regression models (Table 3), with CAP as the continuous dependent variable, waist circumference emerged as an independent predictor of steatosis.

In Model A (including MUAC), waist circumference remained significantly associated with CAP (B = 1.47, *p* = 0.004), whereas SHBG and MUAC were not significant predictors. Similarly, in Model B (including TSF), waist circumference remained independently associated with CAP (B = 1.54, *p* = 0.002), while SHBG and TSF were not significant.

These models explained approximately 18% of the variance in CAP (adjusted R^2^ 0.177–0.184).

### 3.5. Effect of Cardiometabolic Risk-Factor Accumulation on Hepatic Steatosis

The number of cardiometabolic risk factors was associated with a graded increase in hepatic steatosis burden (Table 4). Mean CAP increased from 231.3 ± 35.2 dB/m among women with two risk factors to 262.9 ± 50.0 dB/m among those with three or more factors, while the prevalence of CAP-defined steatosis increased from 28.9% to 61.4%. Each additional cardiometabolic risk factor was associated with a 30.59 dB/m increase in continuous CAP (95% CI 17.33–43.85; *p* < 0.001) and 3.38-fold higher odds of CAP-defined steatosis (95% CI 1.59–7.20; *p* = 0.002). The association remained significant after excluding the three women with only one risk factor (≥3 versus 2 factors; Welch’s *p* = 0.004). These findings indicate that hepatic steatosis burden deteriorates progressively with the absolute number of accumulated cardiometabolic risk factors.

### 3.6. Incremental Predictive Value of Waist Circumference Beyond BMI for CAP-Defined Steatosis

Incremental modelling analyses evaluating the predictive value of central adiposity beyond general obesity are presented in Table 5. In models assessing CAP-defined steatosis, BMI ≥ 30 kg/m^2^ was independently associated with steatosis in Model 1 (OR 13.07, 95% CI 1.57–108.41, *p* = 0.017). Following the addition of waist circumference ≥ 88 cm in Model 2, waist circumference remained independently associated with CAP-defined steatosis (OR 2.77, 95% CI 1.08–7.13, *p* = 0.035), whereas the association with BMI was attenuated and no longer statistically significant (*p* = 0.070). The addition of waist circumference improved model fit (Nagelkerke R^2^ 0.140 vs. 0.202; Δχ^2^ = 4.59, *p* = 0.032) and increased overall classification accuracy from 62.8% to 66.3%, supporting the incremental predictive value of central adiposity beyond BMI alone. Collectively, these findings suggest that assessment of central adiposity may provide clinically relevant information beyond BMI alone when evaluating steatosis risk in postmenopausal women.

## 4. Discussion

In this cohort of postmenopausal women, hepatic steatosis and MASLD were closely linked to adiposity and to the burden of coexisting cardiometabolic abnormalities. Among all anthropometric and hormonal markers examined, waist circumference emerged as the most consistent independent correlate of hepatic steatosis. Women with CAP-defined steatosis exhibited higher BMI and waist circumference and lower SHBG concentrations, with only borderline differences in peripheral adiposity (MUAC and TSF) and no significant difference in androgen levels between groups. Across multivariable analyses, waist circumference remained independently associated with CAP-defined steatosis, and associations with SHBG, MUAC, and TSF were attenuated after adjustment. Beyond any single adiposity measure, the burden of hepatic steatosis increased progressively with the number of accumulated cardiometabolic risk factors: each additional factor was associated with a stepwise rise in CAP and in the odds of steatosis. Notably, because almost all women with steatosis already met at least one cardiometabolic criterion, MASLD and CAP-defined steatosis were largely coincident in this population. Age, years since menopause, blood pressure, and lifestyle-related factors were not independently associated with steatosis, suggesting that adiposity-related metabolic dysfunction and the accumulation of cardiometabolic risk factors, rather than hormonal or behavioural factors, predominantly drive the MASLD phenotype in postmenopausal women.

These findings support the concept that fat distribution may be particularly relevant in addition to overall adiposity in determining hepatic steatosis risk after menopause. Although BMI remains a well-established risk factor for fatty liver disease, it may inadequately capture the redistribution of adipose tissue that occurs during the menopausal transition [23]. In our study, BMI demonstrated the strongest univariate correlation with CAP among the anthropometric measures, although the metabolic markers insulin and HOMA-IR correlated more strongly still, and waist circumference retained an independent association after multivariable adjustment; both observations are consistent with a contribution of metabolically active abdominal fat to hepatic steatosis in postmenopausal women. These anthropometric associations should not, however, be overinterpreted in isolation: the clearest signal in our data was that the burden of hepatic steatosis rose progressively with the number of coexisting cardiometabolic risk factors, indicating that steatosis in this population reflects the accumulation of metabolic dysfunction rather than any single adiposity measure. These findings help address recent knowledge gaps highlighted by Ren et al. regarding the limited availability of clinically phenotyped postmenopausal MASLD cohorts and the contribution of adiposity and metabolic factors to hepatic steatosis risk in this population [11].

The inverse association between SHBG and hepatic steatosis observed in our univariate analyses aligns with extensive evidence linking low SHBG levels to fatty liver disease [24]. SHBG is synthesised in the liver and has increasingly been recognised as a marker of metabolic dysfunction and insulin resistance [25]. Prior population-based, cross-sectional, and longitudinal studies have demonstrated associations between lower SHBG concentrations and greater liver fat accumulation, as well as the progression of steatotic liver disease [25,26,27,28,29,30]. However, in the present study, the association between SHBG and CAP was attenuated after adjustment for adiposity measures, suggesting that lower SHBG concentrations may largely reflect underlying metabolic dysfunction and central obesity rather than exerting an independent effect [31].

Similarly, MUAC and TSF were positively associated with CAP in correlation analyses and were marginally higher among women with CAP-defined steatosis. These findings are biologically plausible, as both measures reflect peripheral adiposity and have previously been associated with obesity-related metabolic risk [32,33,34,35]. Analysis of NHANES 2017–2018 data demonstrated that MUAC is associated with hepatic steatosis in population-based studies using CAP assessment [34]. Our results demonstrate that both measures were associated with hepatic steatosis in univariate analyses, supporting the potential relevance of peripheral adiposity in metabolic liver disease at the steatosis stage.

Importantly, our findings highlight the complementary roles of general and central adiposity within the MASLD framework in postmenopausal women [4]. BMI was strongly associated with hepatic steatosis in the univariate analysis, whereas waist circumference was independently associated with CAP-defined steatosis [36]. Among the individual anthropometric and hormonal measures, waist circumference was the most consistent, but its effect was modest and the peripheral and hormonal markers were attenuated after adjustment, so no single measure should be regarded as the principal driver of steatosis in this population [37,38]. Our findings are in line with the NHANES 2017–2020 study [38], which found that higher weight-adjusted waist circumference, a marker of central adiposity, was independently associated with hepatic steatosis and fibrosis in women, consistent with our findings that central adiposity predicts hepatic steatosis in postmenopausal women better than BMI or peripheral markers.

Taken together, these findings support systematically counting the coexisting cardiometabolic risk factors, alongside simple adiposity measures such as waist circumference, when evaluating steatosis risk in postmenopausal women, particularly given the increasing recognition of MASLD as part of a broader metabolic disease spectrum [11].

Several limitations should be acknowledged. The cross-sectional design precludes causal inference and limits interpretation regarding temporal relationships between adiposity, hormonal changes, and liver disease. The modest sample size may have reduced statistical power to detect weaker associations, particularly for hormonal variables and peripheral adiposity markers. HOMA-IR was not included as a confounder because insulin resistance is likely to lie on the causal pathway between adiposity and MASLD; adjustment for it could therefore constitute over-adjustment and attenuate the estimated associations. In addition, although transient elastography with CAP is a validated non-invasive technique for the assessment of steatosis, liver biopsy remains the reference standard. Residual confounding cannot be excluded despite multivariable adjustment. Finally, the study population was derived from a tertiary menopause clinic and may not fully represent the general postmenopausal population [11].

## 5. Conclusions

In this postmenopausal cohort, the prevalence of MASLD (44.8%) closely approached that of CAP-defined hepatic steatosis (46.0%), because all but one woman with steatosis already carried at least one cardiometabolic risk factor; in this population, therefore, steatosis and MASLD are largely coincident, and the cardiometabolic qualifier adds little discriminatory value. More importantly, hepatic steatosis burden deteriorated progressively with the absolute number of accumulated cardiometabolic risk factors, with each additional factor associated with a stepwise rise in controlled attenuation parameter and in the odds of steatosis. These findings suggest that, after menopause, the number of coexisting cardiometabolic abnormalities is a key determinant of the severity of steatotic liver disease, and support longitudinal studies to clarify how the accumulation of metabolic risk factors, alongside hormonal changes, drives the progression of MASLD in this population.

## Figures and Tables

**Table 1:** (**a**) Clinical, anthropometric, and metabolic characteristics of postmenopausal women according to CAP-defined hepatic steatosis status. (**b**) Hormonal parameters of postmenopausal women, stratified according to the presence or absence of MASLD diagnosis.

(**a**)				
**Variable**	**Overall** **(*n* = 87)**	**CAP < 248** **(*n* = 47)**	**CAP ≥ 248** **(*n* = 40)**	***p*-Value**
Age at visit (years)	64.4 ± 7.5	64.1 ± 8.4	64.7 ± 6.2	0.705
Years since menopause	14.7 ± 7.6	14.9 ± 7.5	14.5 ± 7.8	0.794
BMI (kg/m^2^)	25.8 ± 4.1	24.5 ± 3.1	27.3 ± 4.7	0.003
Waist circumference (cm)	88.0 ± 11.8	84.3 ± 9.9	92.4 ± 12.5	0.001
Systolic BP (mmHg)	115.0 ± 23.3	111.5 ± 20.6	119.0 ± 25.7	0.148
Diastolic BP (mmHg)	71.3 ± 13.9	68.8 ± 12.3	74.2 ± 15.2	0.083
Triceps skinfold (mm)	28.7 ± 6.1	27.6 ± 5.8	30.2 ± 6.3	0.069
Mid-upper arm circumference (cm)	30.9 ± 4.3	30.0 ± 4.4	31.9 ± 4.1	0.053
Glucose (mg/dL)	104.3 ± 12.8	100.6 ± 10.7	108.8 ± 13.7	0.003
Insulin (μIU/mL)	7.9 (6.0–12.4)	6.3 (4.5–9.2)	9.8 (7.5–24.3)	0.002
HOMA-IR	2.07 (1.63–3.47)	1.6 (1.0–2.4)	3.1 (1.8–6.3)	0.002
Current smoking, *n* (%)	12 (13.8)	9 (19.1)	3 (7.5)	0.116
CAP score (dB/m)	246.2 ± 48.0	212.1 ± 31.4	286.2 ± 29.6	<0.001
MASLD (CAP-defined), *n* (%)	39 (44.8%)	—	—	—
**Cardiometabolic risk factors, *n* (%)**				
0 risk factors	2 (2.3%)	1 (2.1%)	1 (2.5%)	0.012
1 risk factor	3 (3.4%)	2 (4.3%)	1 (2.5%)	
2 risk factors	38 (43.7%)	27 (57.6%)	11 (27.5%)	
≥3 risk factors	44 (50.6%)	17 (36.2%)	27 (67.5%)	
(**b**)				
**Parameter**	**No MASLD** **(*n* = 48)**	**MASLD** **(*n* = 39)**		***p*-Value**
Testosterone (nmol/L) †	0.3 (0.2–0.5)	0.37 (0.1–0.7)		0.850
SHBG (nmol/L)	83.5 ± 27.6	62.2 ± 39.4		0.009

Values are mean ± SD or median (25th–75th percentile) or *n* (%). Groups (CAP < 248 vs. ≥248) were compared using independent-samples t-tests for continuous variables and Chi-square or Fisher’s exact tests for categorical variables. Abbreviations: BMI, body mass index; BP, blood pressure; CAP, controlled attenuation parameter; MASLD, metabolic dysfunction-associated steatotic liver disease. Notes: Column headings give the maximum group sizes. Where data were incomplete, analyses used all available observations for the variable concerned. CAP ≥ 248 dB/m defined steatosis. MASLD was defined as steatosis with ≥1 cardiometabolic risk factor (obesity, dyslipidaemia, hypertension, or glucose abnormalities). Statistical significance was set at *p* < 0.05. MASLD was defined as CAP-defined steatosis (CAP ≥ 248 dB/m) in the presence of at least one cardiometabolic risk factor. CAP-defined steatosis was defined as CAP ≥ 248 dB/m. † Testosterone is shown as the untransformed concentration (nmol/L); because its distribution was skewed, the between-group comparison was performed on log-transformed values. Abbreviations: SHBG, sex hormone-binding globulin. Notes: Groups defined by MASLD status and by CAP-defined steatosis differed by a single participant, and comparisons using either definition gave materially the same result. Column headings give the maximum group sizes. Where data were incomplete, analyses used all available observations for the variable concerned. Comparisons between groups were performed using independent-samples t-tests or non-parametric Mann–Whitney. Statistical significance was set at the level of *p* < 0.05.

**Table 2:** Correlations of liver steatosis (CAP) with clinical, hormonal, and anthropometric parameters.

Variable	r Coefficient	*p*-Value
Age (years)	−0.001	0.990
Years since menopause (years)	−0.012	0.909
Body mass index (kg/m^2^)	0.508	<0.001
Waist circumference (cm)	0.433	<0.001
Waist-to-hip ratio	0.175	0.126
Systolic blood pressure (mmHg)	0.110	0.308
Diastolic blood pressure (mmHg)	0.150	0.165
Glucose (mg/dL)	0.317	0.003
Insulin (μIU/mL)	0.609	<0.001
HOMA-IR	0.636	<0.001
SHBG (nmol/L)	−0.254	0.030
Mid-upper arm circumference (cm)	0.350	0.002
Triceps skinfold thickness (mm)	0.351	0.002

Values are Pearson correlation coefficients (r) with corresponding two-sided *p*-values. Analyses were performed using pairwise complete observations. Abbreviations: CAP, controlled attenuation parameter; SHBG, sex hormone-binding globulin. Statistical significance was set at *p* < 0.05.

**Table 3:** Multivariable linear regression model predicting liver fat (CAP). Results are presented as unstandardized regression coefficients (B) with corresponding *p*-values. The model was constructed using the enter method, including variables selected a priori based on biological plausibility and univariable associations. CAP (dB/m) was used as a continuous dependent variable. Statistical significance was set at *p* < 0.05.

Variable	Adjusted R^2^	B Coefficient (95% CI)	*p*-Value
Model A			
Waist circumference (cm)	0.184	1.47 (0.48 to 2.46)	0.004
SHBG (nmol/L)		−0.10 (−0.38 to 0.18)	0.476
Mid-upper arm circumference (cm)		1.31 (−1.34 to 3.96)	0.328
Model B			
Waist circumference (cm)	0.177	1.542 (0.58 to 2.50)	0.002
SHBG (nmol/L)		−0.084 (−0.39 to 0.22)	0.581
Triceps skinfold (mm)		0.619 (−1.22 to 2.45)	0.504

B, unstandardized regression coefficient; CAP, controlled attenuation parameter; SHBG, sex hormone-binding globulin. CAP (dB/m) was analysed as a continuous dependent variable. Variables were entered simultaneously (multivariable linear regression). Adjusted R^2^ is reported as the model fit statistic and represents the proportion of variance explained by the model after accounting for the number of predictors. Statistical significance was set at *p* < 0.05. Model A included waist circumference, SHBG, and mid-upper arm circumference; Model B included waist circumference, SHBG, and triceps skinfold thickness.

**Table 4:** Increase in hepatic steatosis with cardiometabolic risk-factor accumulation in postmenopausal women (*n* = 87).

Analysis	Comparison	Effect Estimate (95% CI)	*p*-Value	Model Performance
Logistic regression	Odds of CAP-defined steatosis per additional risk factor	OR 3.38 (1.59–7.20)	0.002	Nagelkerke (R^2^ = 0.175); accuracy 65.9%
Logistic regression	≥3 versus <3 risk factors	OR 3.84 (1.55–9.50)	0.004	Nagelkerke (R^2^ = 0.134); accuracy 65.9%
Linear regression	Increase in CAP per additional risk factor	B 30.59 dB/m (17.33–43.85)	<0.001	adjusted R^2^ 0.193
CAP group comparison	2 versus ≥3 risk factors, 231.3 ± 35.2 dB/m vs. 262.9 ± 50.0 dB/m	Mean difference 31.57 dB/m (8.98–54.15)	0.004 ^1^	—

^1^ Welch’s comparison after excluding the three women with only one cardiometabolic risk factor. Values are mean ± standard deviation or percentages, as appropriate. CAP-defined hepatic steatosis was defined as CAP ≥ 248 dB/m. B represents the increase in continuous CAP per additional cardiometabolic risk factor. OR, odds ratio; CI, confidence interval; CAP, controlled attenuation parameter. Abbreviations: CAP, controlled attenuation parameter; OR, odds ratio; CI, confidence interval; SD, standard deviation. Statistical significance was set at *p* < 0.05.

**Table 5:** Incremental predictive value of waist circumference beyond body mass index (BMI) for controlled attenuation parameter (CAP)-defined hepatic steatosis. Multivariable logistic regression models evaluating the association of general (BMI ≥ 30 kg/m^2^) and central adiposity (waist circumference ≥ 88 cm) with CAP-defined hepatic steatosis (CAP ≥ 248 dB/m). Model 1 includes BMI alone, while Model 2 includes both BMI and waist circumference. Model performance is reported using Nagelkerke R^2^ and overall classification accuracy.

Model	Predictor	OR (95% CI)	*p*-Value	Nagelkerke R^2^	Overall Accuracy (%)
Model 1	BMI ≥ 30 kg/m^2^	13.07 (1.57–108.41)	0.017	0.140	62.8
Model 2	BMI ≥ 30 kg/m^2^	7.50 (0.85–66.13)	0.070	0.202	66.3
	Waist ≥ 88 cm	2.77 (1.08–7.13)	0.035		

Values are presented as odds ratios (OR) with 95% confidence intervals (CIs). Logistic regression analyses were performed using the enter method. CAP-defined steatosis was defined as CAP ≥ 248 dB/m. BMI ≥ 30 kg/m^2^ and waist circumference ≥ 88 cm were analysed as binary variables. Nagelkerke R^2^ is reported as the model fit statistic and reflects the model’s explanatory power. Wide confidence intervals reflect the limited number of participants meeting obesity and steatosis criteria. Overall accuracy represents the proportion of correctly classified cases using a probability cut-off of 0.5. Addition of waist circumference significantly improved model fit (Δχ^2^ = 4.59, *p* = 0.032). Statistical significance was defined as *p* < 0.05.

## Data Availability

Data is available by the corresponding author upon a reasonable request.

## Abbreviations

The following abbreviations are used in this manuscript:

ALT	Alanine Aminotransferase
AST	Aspartate Aminotransferase
BMI	Body Mass Index
BP	Blood Pressure
CAP	Controlled Attenuation Parameter
CI	Confidence Interval
dB/m	Decibels per Metre
GGT	Gamma-Glutamyl Transferase
HOMA-IR	Homeostatic Model Assessment of Insulin Resistance
IPAQ	International Physical Activity Questionnaire
IQR	Interquartile Range
LnTestosterone	Log-transformed Testosterone
MASLD	Metabolic Dysfunction-Associated Steatotic Liver Disease
MET	Metabolic Equivalent of Task
MUAC	Mid-Upper Arm Circumference
OR	Odds Ratio
R^2^	Coefficient of Determination
SD	Standard Deviation
SHBG	Sex Hormone-Binding Globulin
SPSS	Statistical Package for the Social Sciences
TSF	Triceps Skinfold Thickness
WHO	World Health Organisation
B	Unstandardized Regression Coefficient
χ^2^	Chi-square Test
